# Landslide susceptibility mapping in an area of underground mining using the multicriteria decision analysis method

**DOI:** 10.1007/s10661-018-7085-5

**Published:** 2018-11-14

**Authors:** Deniz Arca, Hakan Ş. Kutoğlu, Kazimierz Becek

**Affiliations:** 1 Department of Izmir Vocational School, Dokuz Eylül University, Izmir, Turkey; 2 Faculty of Engineering, Department of Geomatics Engineering, Bülent Ecevit University, Zonguldak, Turkey; 3 Geoengineering, Mining and Geology, Wroclaw University of Science & Technology, Wroclaw, Poland

**Keywords:** Landslide susceptibility, Underground mining, Subsidence, Multicriteria decision analysis, GIS, Kozlu, Turkey

## Abstract

Landslides are geomorphological phenomena that affect anthropogenic and natural features on the Earth’s surface. Many previous studies have identified several factors that have contributed to landslides. Among these factors are physical characteristics, such as slope, aspect, and land cover, of Earth’s surface. Moreover, landslides can be triggered by human activities such as underground mining. This study aims to identify landslide susceptibility areas by analyzing landslide-related factors, including land subsidence triggered by underground mining. The area of interest was Kozlu, Turkey, where underground mining has been in progress for the past 100 years. Thus, to identify landslide risk zones, the multicriteria decision analysis method, together with the analytical hierarchy method, was used. The datasets included were topography, land cover, geological settings, and mining-induced land subsidence. The spatial extent of land subsidence was estimated using a previously published model. A landslide susceptibility map (LSM) was developed using a purposely developed GIS-based software. The results were compared with a terrain deformation map, which was developed in a separate study using the differential synthetic aperture radar interferometry (DInSAR) technique. The results showed a substantial correlation between the LSM and DInSAR map. Furthermore, it was found that ~ 88% of the very high and high landslide risk areas coincided with location of the past landslide events. These facts suggest that the algorithm and data sources used were sufficient to produce a sufficiently accurate LSM, which may be used for various purposes such as urban planning.

**Electronic supplementary material:**

The online version of this article (10.1007/s10661-018-7085-5) contains supplementary material, which is available to authorized users.

## Introduction

Underground mining disturbs the Earth’s natural balance and generally causes terrain subsidence. Such subsidence is common in Kozlu, a hard coal basin in Zonguldak, Turkey (Akcin 1995). In this region, underground coal extraction has been performed for > 150 years. During this period, ~ 400 million tons of hard coal has been extracted. Mining-induced terrain subsidence has had adverse effects on structures in urban areas in the mining region such as causing damage to property (Abdikan 2012). In the area of interest (AOI), landslides and sinkholes are common because of the karstic properties of rocks that dominate the AOI’s geology. Failure to observe basic construction rules that are applicable to buildings in mining areas has aggravated hazards to on-the-ground structures in the AOI. Consequently, almost every year, landslide-related incidents have led to property damage and even loss of life (Akcin 1995). This adverse situation triggered a range of mitigation measures by local authorities such as landslide susceptibility studies (Gokceoglu and Aksoy 1996; Karakaya 2003; Suzen and Doyuran 2004; Ercanoglu et al. 2004; Corekcioglu 2004; Yesilnacar and Topal 2005).

To estimate terrain subsidence, various methods, both empirical and theoretical, can be used with various success rates. These methods could be used to develop landslide susceptibility maps (LSMs) for certain locations that have specific geomorphological and other conditions. The Kozlu mining area in Zonguldak is the oldest coal mining area in Turkey; it is the only mine in which certain production shafts are under the sea bed. Recently, coal production in the area has decreased; however, the government has taken measures that are aimed at reversing this trend or at least to stabilize the level of coal production.

Our study aims to develop a LSM for the Kozlu mining area in Zonguldak using the multicriteria decision analysis (MCDA) method. The input variables include slope, aspect and elevation, rock type, land use, and distance to major roads. Moreover, estimated land subsidence based on a model described by Peng (1992) was included. Each variable contributed to landslides or subsidence susceptibility to some extent. Thus, we established weights for each variable using the weighted linear method (WLM). The LSM for the AOI helped distinguish five risk levels. The map was superimposed on a landslide inventory map, developed by mining authorities, to assess the level of agreement between modeled subsidence and observed surface subsidence. Moreover, the map was compared with a land subsidence map of the AOI, which was developed using differential synthetic aperture radar interferometry (DInSAR) in a separate study (Kutoğlu et al. 2016). We achieved a satisfactory level of agreement between both reference datasets and the LSM. The LSM was developed using a software that was developed using a Python-based script, which was integrated within the ArcGIS (ESRI, Redlands, CA) software package.

## Material and methods

### Study area

**Fig. 1:**
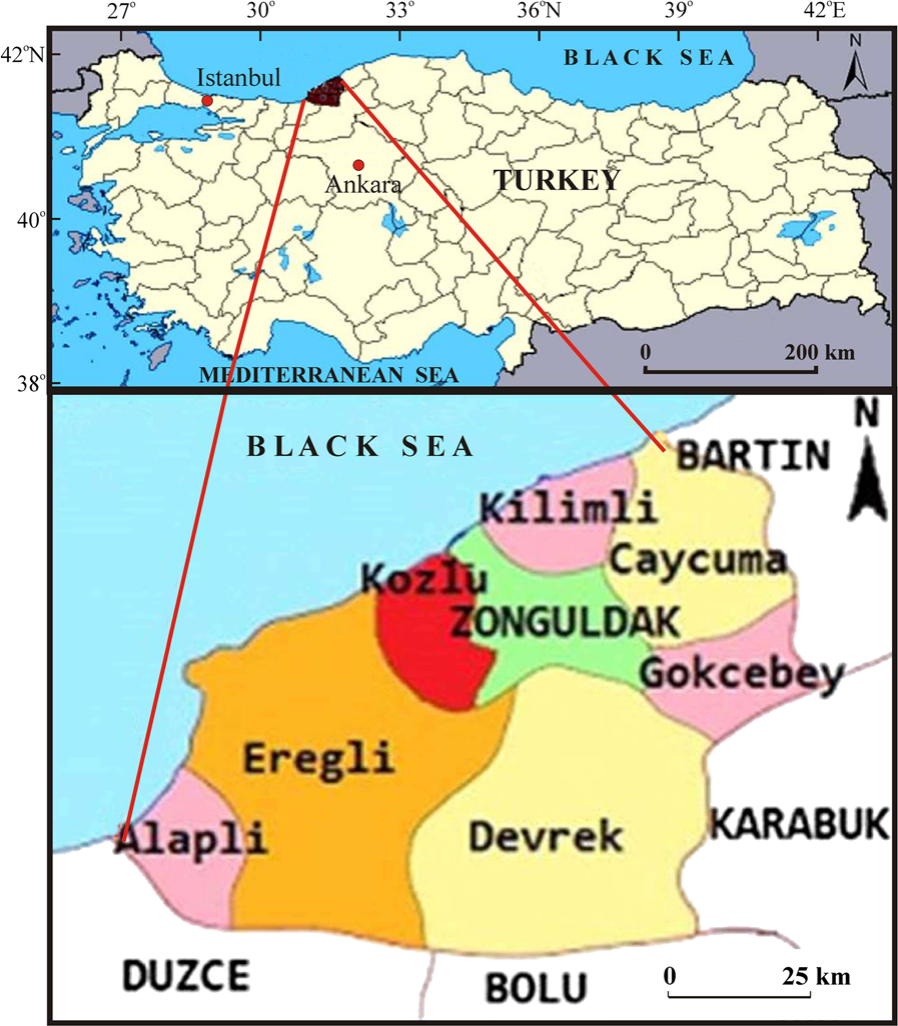
Location map of the study area. (*Source*: modified figure from Can et al. 2012)

Figure 1 shows the AOI with the study area composed of Kozlu County, which is located in Zonguldak municipality in the Republic of Turkey on the Black Sea coast (41° 27′ N, 31° 49′ E). The coverage is an area of ~ 1832 ha, including ~ 1063 ha for urban land use. Note that the study area bordered the cities of Zonguldak, Ereğli, Caycuma, and Beycuma (Can 2011).

The topography of the study area is hilly with locally steep slopes. The terrain elevation varies from a certain range onwards up to 200 m. The top soil comprises weathered rocks of sedimentary and metamorphic origin. The vegetation on extreme slopes is mixed temperate forest, whereas gentle slopes have orchards or gardens growing on them. Urban areas are paved with concrete slabs, and rainwater is drained away through a dense network of concrete channels. Note that the climate type of the study area is oceanic with precipitation evenly distributed throughout the year. Summers are warm and humid, whereas winters are cool and damp. The average temperature in January and February is ~ 6 °C, whereas it is around 21 °C in July and August. The average annual precipitation is ~ 1220 mm, and it is heaviest in the fall and winter. Snowfall is commonly seen from December to March and it lasts for a week or two. The sea water temperature fluctuates between 8 and 20 °C throughout the year. The study area is home to ~ 300,000 people, all of whom reside in dwellings built without any definite design plan (Abdikan 2012). Generally, in these regions, different types of landslide such as flows, rotational slides, and, to a lesser extent, topples are observed (Can 2014).

### Geological conditions in Kozlu, Zonguldak: a hard coal production region

In addition to hard coal, many minerals, such as bauxite, barite, dolomite, limestone, basalt, manganese, quartzite, and schieferton, were found in the study area. Hard coal mining has been conducted in this area since 1848, and coal is one of the primary energy sources for Turkey. Mining operations were conducted at levels of ~ 345–630 m below the ground surface.

The study area comprises various geological units, such as Kozlu, Karadon, and Inalti formations, as well as the Incigez Detritic member, alluvium, present-time beach sand, and landfills. The Kozlu formation comprises conglomerate, mudstone, intercalations of medium-coarse grained sandstone, and coal lithology, with a thickness of ~ 700 m. The Karadon formation has a thickness of 300–400 m and is composed of conglomerate, sandstone, siltstone, claystone, coal, and fireclay. The gray and brown Inalti formation is composed of limestone, sandy limestone, and conglomerate with intercalations of limestone layers that are up to 2–4 m thick (Yergok et al. 1987). The Incigez Detritic member contains conglomerate, sandstone, siltstone, and limestone intercalations with a gray and reddish-brown color (Alan and Aksay 2002). The Kozlu Creek and the Black Sea shoreline are composed of alluvial rocks, with clay, silt, sand, gravel, and blocks.

In certain locations, landslides have occurred and sinkholes have formed. The karstic rock properties of the area were identified as a contributory factor to these problems.

### Data on production panels in Kozlu mines

**Fig. 2:**
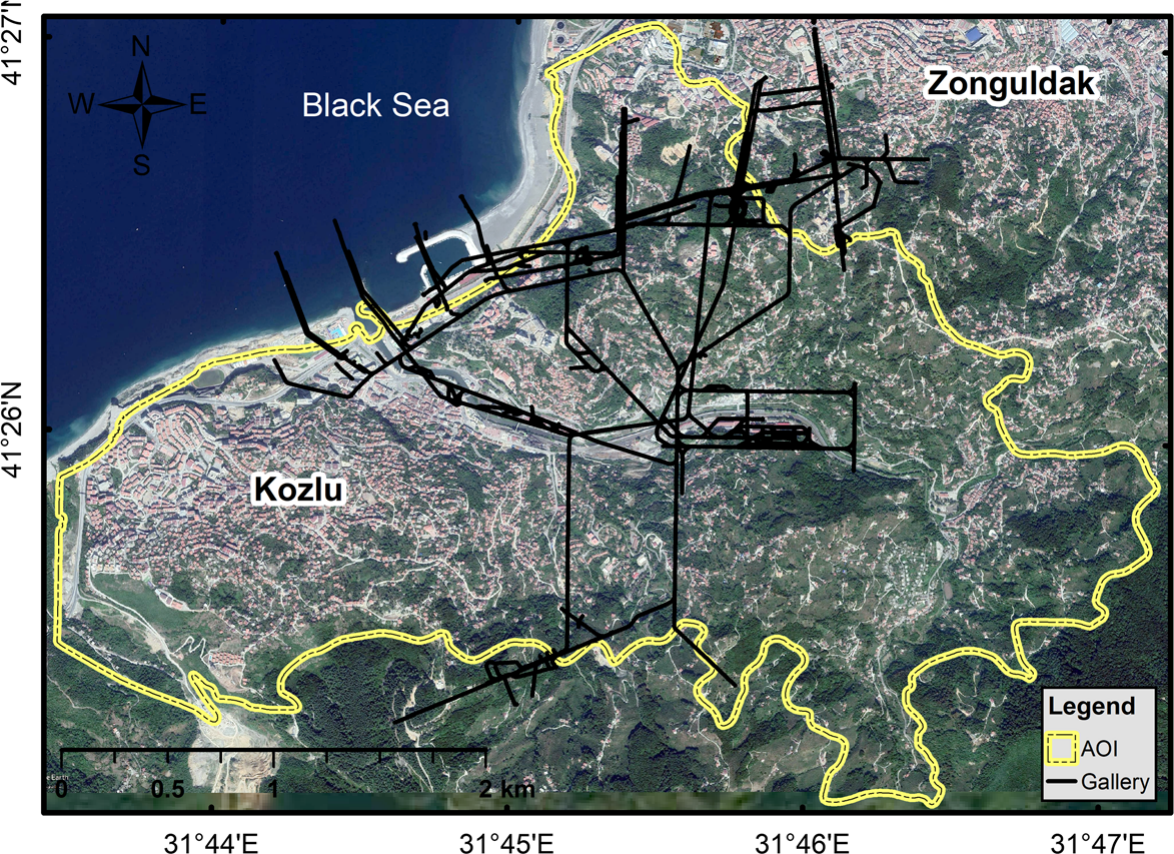
Coal production galleries in Kozlu Basin. (*Source*: Can et al. 2012, Google Earth^®^)

The Kozlu Institute is a member of the Turkish Hard Coal Authority, which is responsible for mining in the Kozlu area. Figure 2 shows the location of mining galleries in the background of a satellite image (Can et al. 2012).

At the Kozlu mines, mining is conducted using a longwall system. Production panels had a sloping structure with new production panels having a depth of ~ 450 m under the Kozlu harbor. Table 1 presents data on the dimensions of these production panels. The data include slope, thickness, width, panel length, and expected maximum subsidence, according to charts and tables of the Subsidence Engineers Handbook (NCB 1975).

**Table 1:** Key quantitative characteristics of the extraction panels in Kozlu. (Source: CMD 2018)

Panel	Slope (°)	Thickness (m)	Width (m)	*H*_l_	*γ*_l_^1)^	Horizontal length (m)	Subsidence (m)
				*H*_m_	*γ*_m_		
				*H*_u_	*γ*_u_		
				(m)	(°)		
New mining panel 1	10	2	155	400	45	340	1.77
				390	81		
				380	68		
New mining panel 2	45	3	240	440	28	220	1.91
				430	61		
				420	85		
New mining panel 3	65	2.5	55	485	32	200	0.95
				430	67		
				380	80		
New mining panel 4	65	2.5	105	560	32	110	0.95
				520	67		
				485	80		
New mining panel 5	30	2	240	560	30	280	1.56
				520	64		
				485	82		
New mining panel 6	30	3	115	560	30	240	2.34
				520	64		
				485	82		
Old mining panel 1	35	3	125	560	28	250	2.21
				520	63		
				485	83		
Old mining panel 2	70	3	50	560	35	510	0.92
				520	70		
				485	78		

^1^The index letter *l*, *m* and *u* of the subsidence border angles (*γ*) indicates the lowest, middle and upper point of panel, respectively

### Subsidence factors

The selection of relevant site characteristics is a key factor in landslide and terrain subsidence studies (Mazman 2005). In the present study, the following factors were selected: slope, aspect, elevation, rock type (lithology), land use, and distance from roads.

**Fig. 3:**
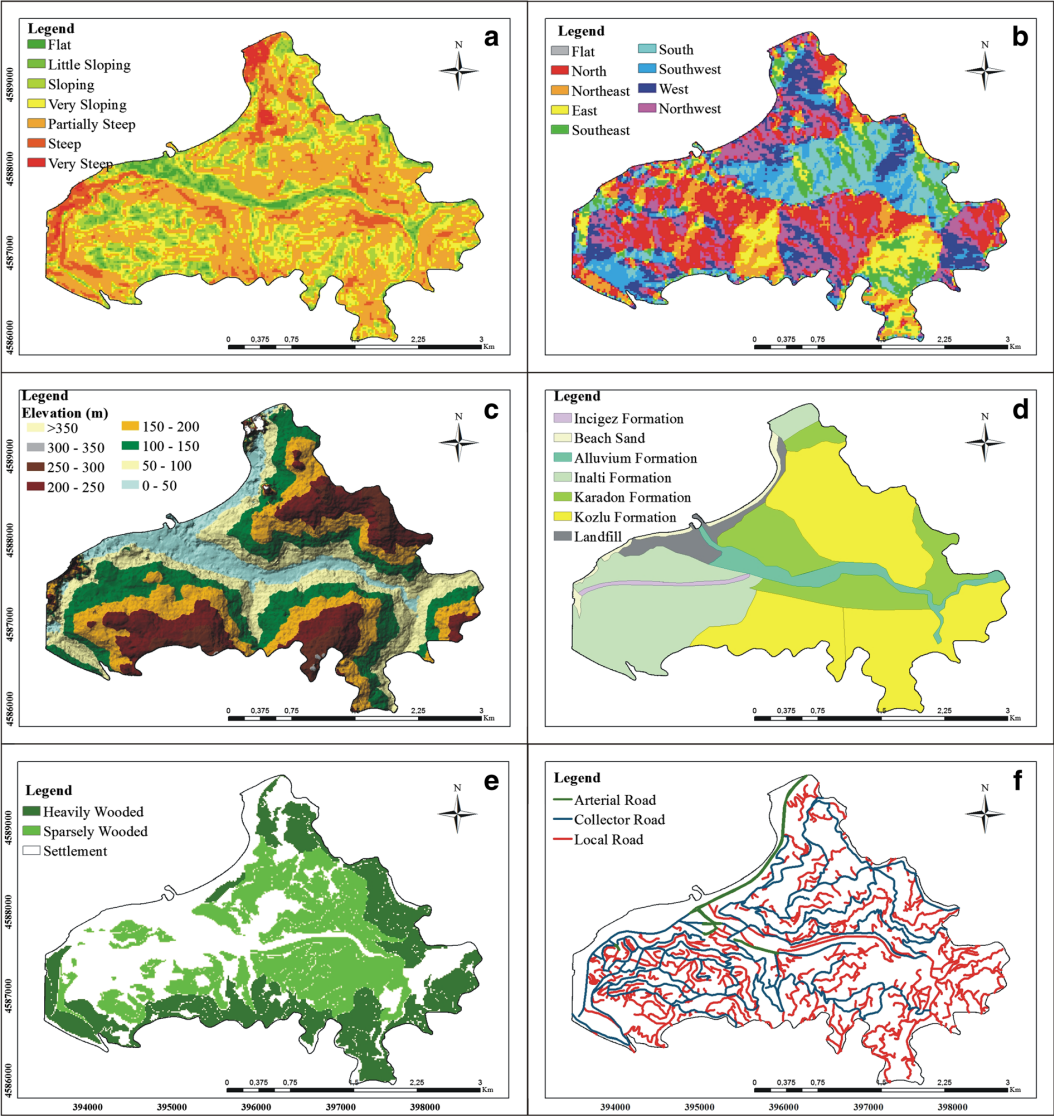
Landslide susceptibility parameters used in this study. **a** Slope, **b** aspect, **c** elevation, **d** rock types, **e** land use, and **f** major roads. (Coordinates: UTM zone 36; datum: WGS 84)

The most significant factor controlling landslide susceptibility was the terrain’s slope (Lee and Min 2001; Dai et al. 2001). A slope map was developed based on a 10-m resolution digital elevation model (DEM). The lowest and highest slope was 0° and 83°, respectively. Figure 3a shows the slope map of the AOI. Moreover, as shown in Table 2, the slope was classified into ten classes.

**Table 2:** Slope classes. (Source: Table 7, FAO 2006)

Class	Description	%
01	Flat	0–0.2
02	Level	0.2–0.5
03	Nearly level	0.5–1.0
04	Very gently sloping	1.0–2.0
05	Gently sloping	2–5
06	Sloping	5–10
07	Strongly sloping	10–15
08	Moderately steep	15–30
09	Steep	30–60
10	Very steep	> 60

In this study, an aspect map was produced using a 10-m resolution DEM. The aspect classes are listed in Table 3, whereas Fig. 3b shows the aspect classes over the AOI.

**Table 3:** Aspect classes

Description	Azimuth (°)
Flat	0
North	337.5–22.5
Northeast	22.5–67.5
East	67.5–112.5
Southeast	112.5–157.5
Southwest	202.5–247.5
West	247.5–292.5
Northwest	292.5–337.5

Furthermore, the terrain elevation is frequently included as a parameter for landslide susceptibility studies (Yan et al. 2018). Elevation affects various biotic factors and anthropogenic elements, which can promote a landslide (Dai and Lee 2002). Figure 3c shows a DEM of the AOI in which the elevation varies between 0 and 450 m. Note that the map shows nine elevation classes at 50-m intervals.

As demonstrated by previous landslide susceptibility studies, the lithological makeup of an area is an important factor for the landslide susceptibility of an area (Ayalew et al. 2005; Wang et al. 2009; Dai and Lee 2002; Karsli et al. 2009). Surface water infiltrating various lithological units leads to “sliding tension,” which increases sliding sensitivity (Yilmaz 2009). Figure 3d shows the spatial distribution of major rock formations that were identified in the AOI.

In landslide studies, as land use changes over time because of natural and anthropogenic forces, land use characteristics of an area become important variables. The effects of anthropogenic forces are particularly intense for industrial areas (Yalcin et al. 2011). Moreover, the destruction of vegetation on slopes and land misuse for various purposes contribute towards increase in the occurrence of landslides (Can 2014). Figure 3e shows the land use characteristics of the AOI. The land use data were extracted via photointerpretation of Google Earth^®^ imagery.

Furthermore, a road system may be an anthropogenic landscape feature that significantly alters the natural stability of terrains and may contribute to landslides. Figure 3f shows the road network in the AOI. In the landslide susceptibility analysis, the “road factor” was modeled using a 25-m wide buffer on each side of the road’s centerline.

### Land subsidence estimation

**Fig. 4:**
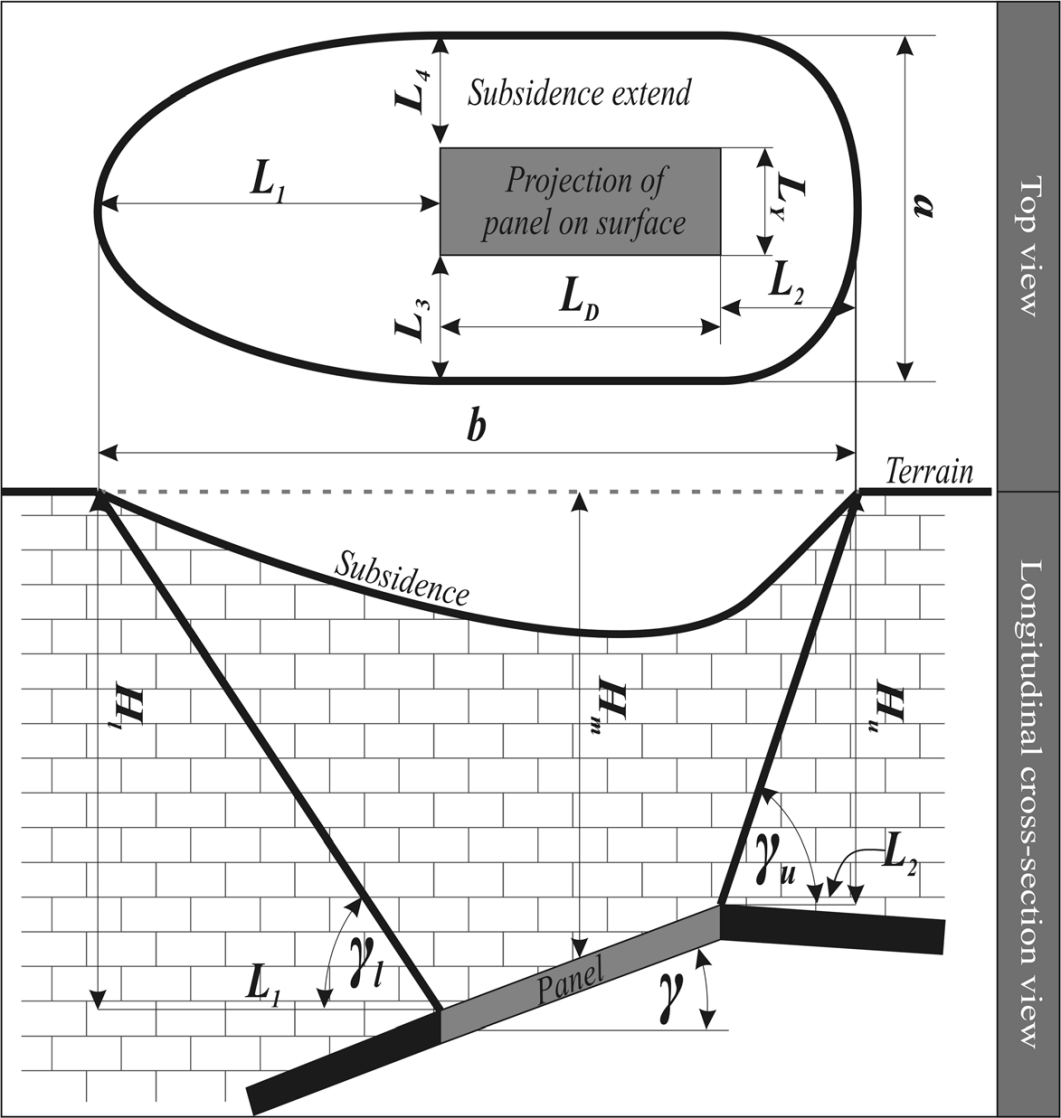
Subsidence affected area. (*Source*: Can et al. 2012)

The extent of mining-induced terrain subsidence can be estimated using a method based on the geometry of geological strata as well as the thickness, slope, and depth of production panels (Akcin 2011). Figure 4 shows a model of subsidence caused by underground mining (Peng 1992). According to this model, the horizontal projection of subsidence area has a quasi-elliptic shape. The shape of quasi-ellipse in relation to horizontal projection of panels was determined using distances *L*_1_, *L*_2_, and *L*_D_, adding up to the major axis of quasi-ellipse; this is denoted in Fig. 4 as *a*. The distances *L*_1_ and *L*_2_ are the “deeper” and “shallower” sections of subsidence zone, respectively, whereas *L*_D_ is the horizontal or projected length of the panel. The minor axis of the subsidence quasi-ellipse, *b*, is the sum of *L*_3_ = *L*_4_ and *L*_Y_, where *L*_3_ and *L*_4_ are equal and calculated in exactly the same manner as *L*_1_ and *L*_2_.

The minor and major axes of quasi-ellipse were calculated from Eqs. (1) and (2):

1 $$ a={L}_{\mathrm{Y}}+2{H}_{\mathrm{m}}\cot {\gamma}_{\mathrm{m},} $$

2 $$ b={L}_{\mathrm{D}}+{H}_{\mathrm{l}}\cot {\gamma}_{\mathrm{l}}+{H}_{\mathrm{u}}\cot {\gamma}_{\mathrm{u}}, $$

where *γ*_l_ and *γ*_u_ are the subsidence limit border angles of the lowest and upper limits of the panel, respectively, *γ*_m_ is the subsidence limit border angle (not shown in Fig. 4) in a direction perpendicular to the longitudinal axis of the panel, and *H*_l_, *H*_m_, and *H*_u_ are the depths of the lower, middle, and upper point of the panel, respectively.

### Application of MCDA to landslide susceptibility mapping

MCDA is a well-known method for solving multivariable and multisolution problems (Malczewski 1999). MCDA is related to mathematical linear programming and the expert system method. Multiple Internet-based open-access sources of information provide details of the MCDA method and its use for solving problems similar to those addressed in the present study. These problems have included wind farm site selection (Szurek et al. 2014), flood susceptibility mapping (Ozturk and Batuk 2011; Kwang and Osei 2017), landfill site selection (Gorevski et al. 2012), and nuclear waste disposal site selection (Carver 1991). In cases of suitable site selection issues, a raster-based version of MCDA is utilized. This approach is termed as the multicriteria evaluation (MCE) method. A key step in the MCE method is to establish a relative weight for each factor, which can be performed in various ways, one of which is the weighted linear combination or scoring method (Saaty 1980). Another method is the pairwise comparison method. A weight for each factor is estimated by comparing the importance of each factor in pairs. The pairwise comparison method contains the following three basic steps: (a) development of a pairwise comparison matrix (PCM), (b) calculation of parameter weights, and (c) estimation of the consistency ratio. The dominance of one factor over the other is scored from 1 to 9, where 1 indicates that both factors are equally important, whereas 9 indicates that the first factor is much more important compared to the second one. It is convenient to record scores in a PCM. The first column of the PCM contains the first member of each pair. The below-diagonal part of the PCM contains the values of the comparison of the second element for each pair with the first one. Hence, the below-diagonal elements of the PCM are reciprocals of the above-diagonal elements. For example, say that element (2,3) = 5; thus, element (3,2) = 1/5. In this study, the PCM estimated is shown in Table 4, and the resulting weighting matrix is shown in Table 5. To verify the consistency of the weights assigned to the factors, the consistency rate (*CR*) was calculated as per Eq. (3):

3 $$ CR=\frac{CI}{RI} $$

where *CI* is the consistency index that provides the consistency discrimination criterion and *RI* is the random index.

**Table 4:** The pairwise comparison matrix

Parameter	Subsidence	Slope	Lithology	Elevation	Aspect	Land use	Distance to road
Subsidence	1	1	4	6	5	7	8
Slope	1	1	1	5	8	7	6
Lithology	1/4	1	1	5	7	7	5
Elevation	1/6	1/5	1/5	1	5	4	3
Aspect	1/5	1/8	1/7	1/5	1	1/3	1/5
Land use	1/7	1/7	1/7	1/4	3	1	1/3
Distance to road	1/8	1/6	1/5	1/3	5	3	1
	2.88	3.63	6.68	17.78	34	29.33	23.53

**Table 5:** The weighting matrix

Parameter	Subsidence	Slope	Lithology	Elevation	Aspect	Land use	Distance to road	Weights
Subsidence	0.35	0.27	0.6	0.34	0.15	0.24	0.3	0.32
Slope	0.35	0.27	0.15	0.28	0.21	0.24	0.23	0.25
Lithology	0.09	0.27	0.15	0.28	0.21	0.24	0.19	0.2
Elevation	0.06	0.05	0.03	0.06	0.15	0.14	0.23	0.1
Aspect	0.07	0.03	0.02	0.01	0.03	0.01	0.01	0.03
Land use	0.05	0.04	0.02	0.01	0.09	0.03	0.01	0.04
Distance to road	0.04	0.05	0.03	0.02	0.15	0.1	0.04	0.06
Sum of weights	1	1	1	1	1	1	1	1

Note that the *CI* is obtained from Eq. (4):

4 $$ CI=\frac{\left(\lambda -n\right)}{\left(n-1\right)} $$

where *λ* is the average of the consistency vector and *n* is the number of criteria.

The *CR* was 0.09, which was lesser compared to the threshold value of 0.1. This indicates that the weights were consistent (Saaty 1977).

In the next step, each input data layer was normalized to an 8-bit raster. Finally, to classify each pixel depending on the level of susceptibility to landslides, the weighted linear combination decision rule method was used. In this method, the weighted factors were summarized to calculate the susceptibility level *S*, which is expressed using Eq. (5) (Voogd 1983):

5 $$ S=\sum \limits_{i=1}^n{w}_i\ast {x}_i, $$

where *w*_*i*_ is the weight of factor *i*, *x*_*i*_ is the criterion score of factor *i*, and *n* is the number of factors.

### Differential synthetic aperture radar interferometry method

The DInSAR method is an air- or space-borne surveying technique for recording temporal changes of the terrain surface because of deformation and land subsidence. It uses high-resolution synthetic aperture radar (SAR) data captured from two different points in space at the same time or in a matter of days. This initial survey, known as a master survey, is compared with a similar type of survey; however, depending on the kinetic properties of the observed phenomenon, sometimes it is performed after the master survey (days, weeks, months, or even years). The sensitivity of the DInSAR land subsidence survey is of the centimeter order (Fletcher 2007). The DInSAR land subsidence survey was previously performed in the AOI in a separate study by one of the co-authors of this study (Kutoğlu et al. 2016). In fact, for the present study, the results of the land subsidence study using the DInSAR method have been used as one of the controls.

## Results

To estimate the extent of land subsidence for each production panel, we used the land subsidence model outlined in “Land subsidence estimation.” The calculation and plotting were performed using a script written in Python, embedded in the ArcGIS (ESRI, Redlands, CA) package. The source code of the Python script was retrieved from an online resource (Subsidence.zip).

Table 6 summarizes the geometric characteristics of the identified subsidence zones. The results are listed in terms of the minor (*a*) and major (*b*) axes of the subsidence quasi-ellipse, along with the *L*_1_ to *L*_4_ parameters, which were used to estimate the axis of the quasi-ellipse (refer to Fig. 4).

**Table 6:** Estimated dimensions of the land subsidence quasi-ellipses for mining panels

Mining panels	*L*_1_ (m)	*L*_2_ (m)	*L*_3_ and *L*_4_ (m)	*A* (m)	*B* (m)	Area (km^2^)
New mining panel 1	400	154	62	464	709	0.26
New mining panel 2	827	37	238	697	1104	0.60
New mining panel 3	776	67	183	565	898	0.40
New mining panel 4	896	86	221	551	1087	0.47
New mining panel 5	969	68	254	787	1278	0.79
New mining panel 6	970	68	254	747	1153	0.68
Old mining panel 1	1053	60	265	780	1238	0.76
Old mining panel 2	800	103	189	889	953	0.66

**Fig. 5:**
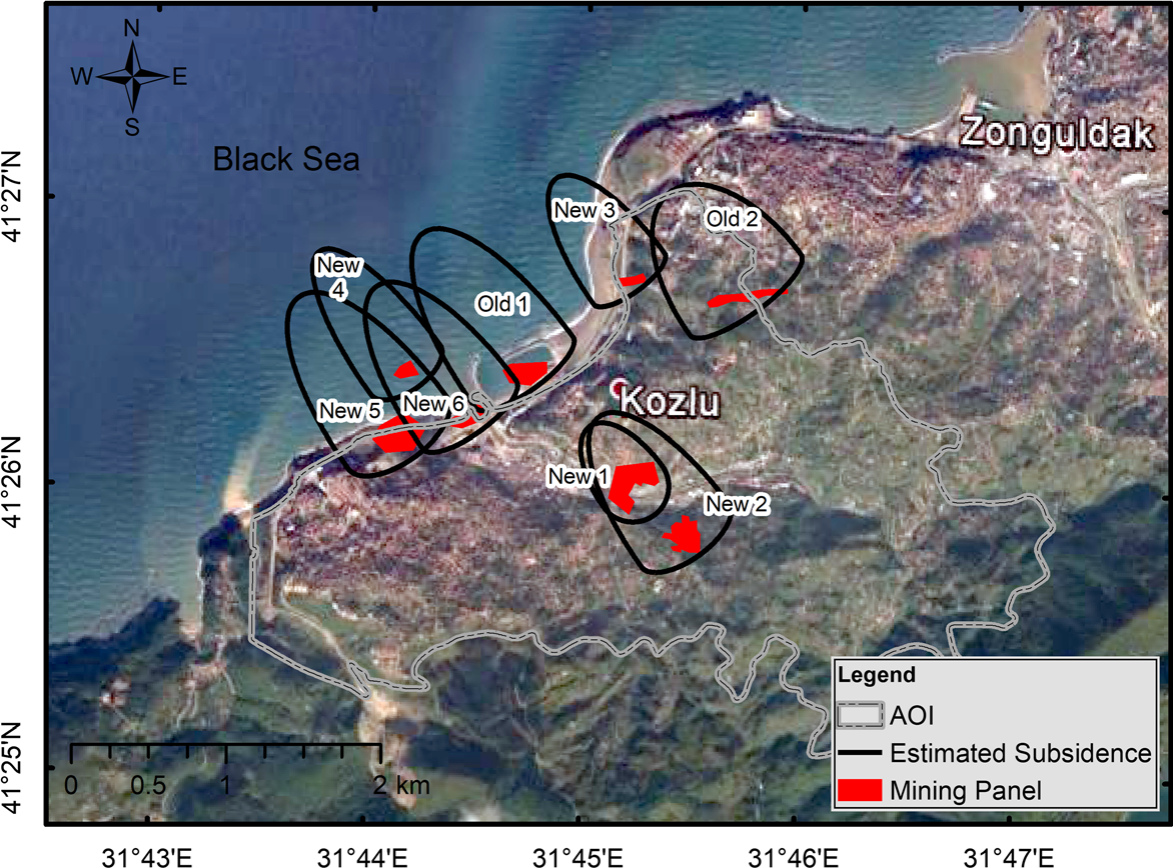
Estimated subsidence ellipses projected onto the location of the mining panels

Figure 5 shows the locations of the subsidence quasi-ellipses, which were projected on the location of the mining panels. A majority of the subsidence zones were offshore (northwestern section of the AOI). However, some zones were onshore in an inhabited section of the AOI. In fact, the total subsidence area was 4.26 km^2^, including a 1.412-km^2^ area that coincided with residential areas with some zones partially overlapping with each other (e.g., New 1 and New 2). Partial overlapping of the zones may further increase the land subsidence effect, which contributes to the landslide risk level.

**Fig. 6:**
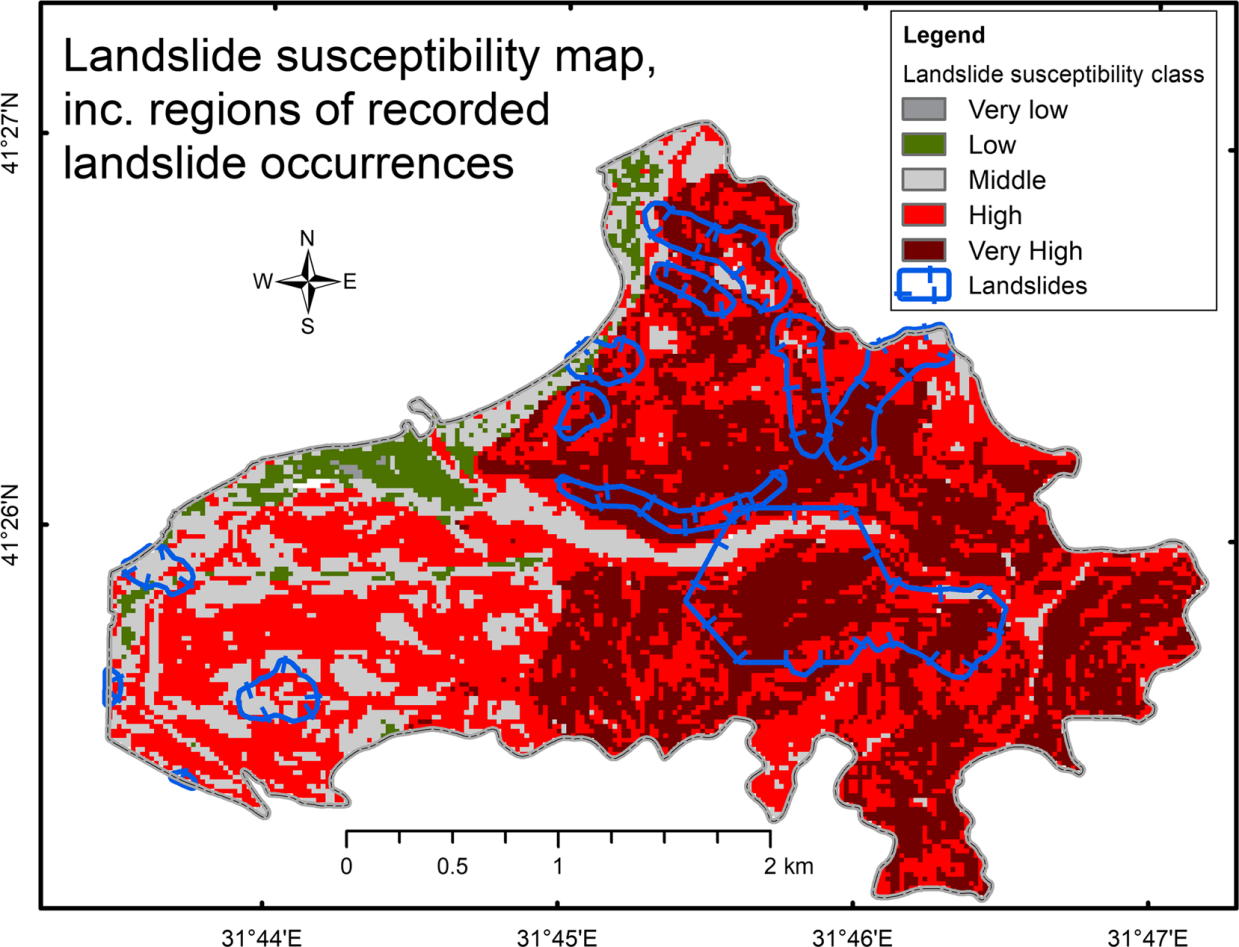
Landslide susceptibility map, including regions of recorded landslide occurrences

For the next data processing step, a landslide susceptibility map was developed, without considering the mining-induced subsidence regions. Figure 6 shows the resulting landslide susceptibility map. As listed in Table 7, the magnitude of landslide susceptibility was divided into five classes, along with the percentage of the AOI for each class.

**Table 7:** Percentage of the landslide susceptibility classes in the AOI

Landslide susceptibility class	Fraction of AOI (%)
Very low	0.1
Low	3.7
Middle	16.5
High	44.8
Very high	34.9
Total	100.0

Furthermore, the landslide susceptibility map (Fig. 6) was overlaid with the recorded landslide occurrences map. Table 8 shows the percentage of the area of the recorded landslide regions for each landslide susceptibility class. The results revealed that 88.2% of the areas in which landslides were recorded belonged to a high or a very high landslide susceptibility class. This figure clearly indicates a high degree of landslide predictability potential for the LSM that was developed for this study.

**Table 8:** Percentage of landslide susceptibility classes present in the recorded landslide regions

Landslide susceptibility class	Fraction of recorded landslide regions (%)
Very low	0.0
Low	0.6
Middle	11.2
High	35.2
Very high	53.0
Total	100.0

**Fig. 7:**
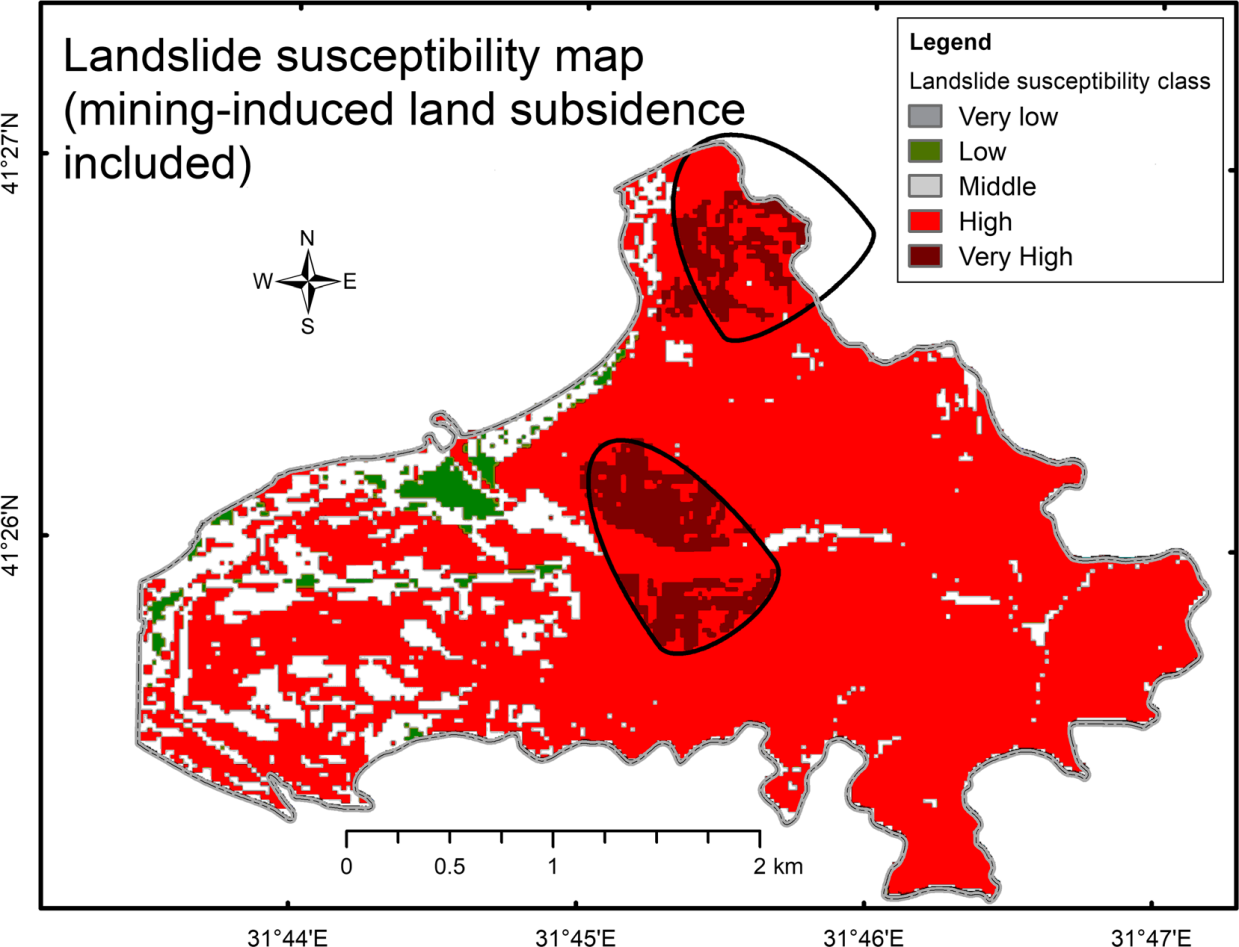
Landslide susceptibility map. The mining-induced land subsidence estimates were included in the calculations

To investigate the impact of mining-induced land subsidence on landslide susceptibility, the subsidence region was included as one of the factors for landslide susceptibility calculations. Figure 7 shows the resulting LSM. Clearly, the highest landslide risk class coincided with the quasi-ellipses of mining-induced land subsidence.

**Fig. 8:**
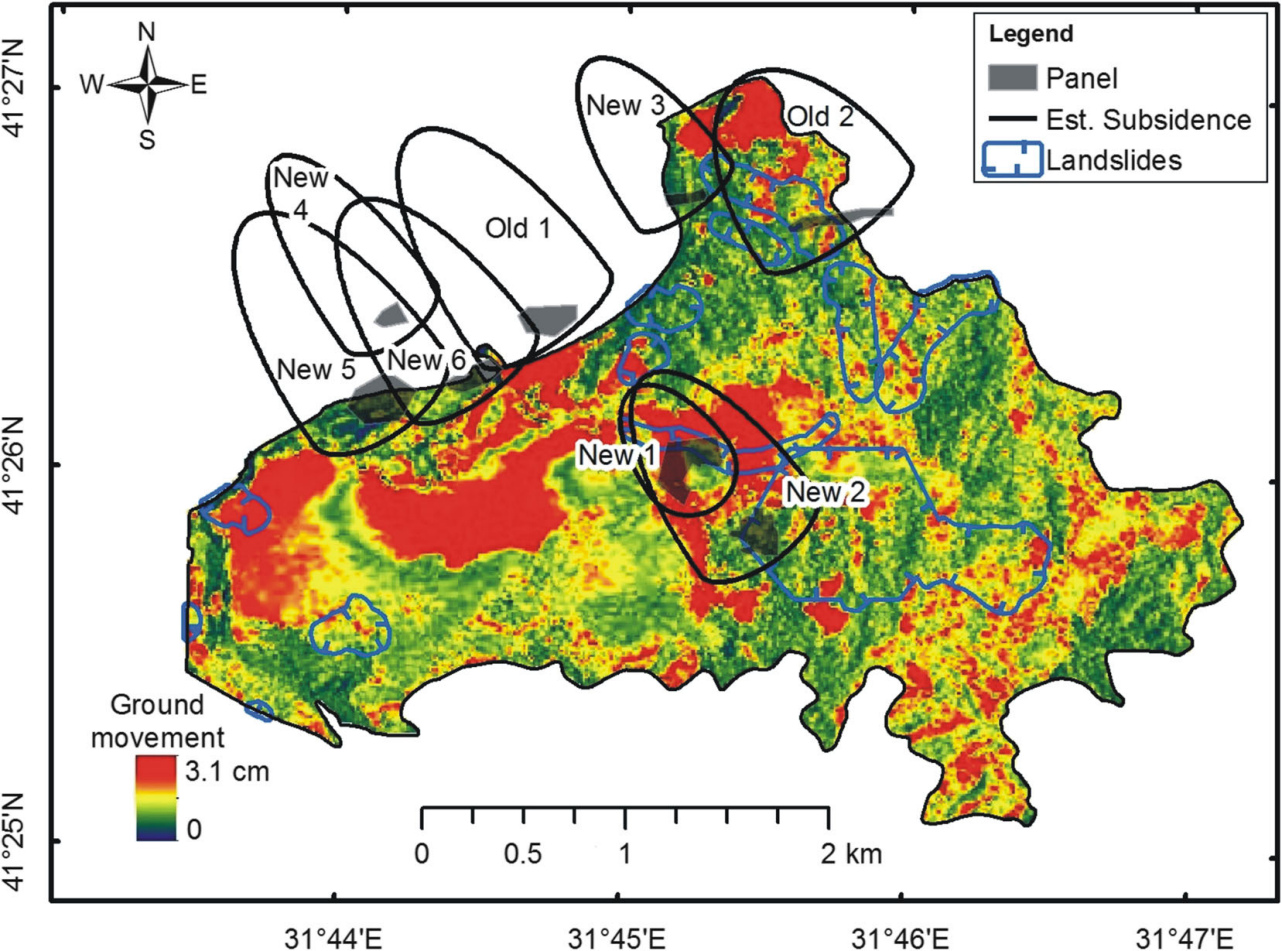
Ground deformation determined using the DInSAR technique based on TerraSAR-X satellite data. (*Source*: Kutoğlu et al. 2016)

To verify these results, the calculated polygons were overlaid on a subsidence map, which was produced using the DInSAR technique (Kutoğlu et al. 2016). Figure 8 shows the DInSAR subsidence map, which was developed based on TerraSAR-X SAR data acquired between March 15, 2011, and September 29, 2011 (Kutoğlu et al. 2016). Note that the map shows the magnitude of terrain deformation in the line of sight direction of the electromagnetic waves incoming from a satellite.

The overall agreement of the calculated subsidence regions with the DInSAR subsidence map is ~ 68% of the total area of the calculated regions.

## Discussion

To predict the exact time and location of a landslide is impossible. However, a large number of studies of landslides have managed to demonstrate links that connect landslide occurrence to particular factors. These studies showed that factors that promote landslides include land cover, slope, aspect, hydrology, soil, and distance from roads (e.g., Dai and Lee 2001, 2002; Ayalew et al. 2004). In the present study, we investigated the impact of an additional factor, i.e., land subsidence caused by underground mining. The available models of land subsidence allow for fairly accurate estimations of spatial extent and magnitude of subsidence. Using these models, we developed a LSM for the Kozlu mining area. The estimates of land subsidence obtained from the map were included in the calculations of a landslide risk map. A Python script was developed to perform all the calculations and plot drawings. To confirm the LSM, it was overlaid with an in situ landslide inventory data and a DInSAR-estimated land subsidence map. A high degree of co-occurrence between both the landslide inventory map (88.2%) and DInSAR map (approx. 68%) was recorded.

The datasets used for the present study were obtained from public records and supplied by the mining company (location and dimensions of mining panels). No information for the currency and accuracy of mining panel’s data was available while acquiring the data for the project. Therefore, some discretion is advised when using the results of this study and, in particular, for land property insurance purposes. However, the unknown level of the currency and accuracy of the input data does not undermine the overall result of the study. This is because the results are above a reasonable level of doubt.

## Conclusions

Based on this study, the following observations can be made:

- The inclusion of mining-induced land subsidence regions as one of the parameters of landslide susceptibility calculations significantly increased the landslide risk level. The magnitude of the impact of this factor depended on the weight assigned to it. A separate study is necessary to investigate the regime for weight selection. Consequently, the results presented should be considered raw indicators only.
- Figure 7 shows a clear correlation between the DInSAR land subsidence map and land subsidence regions identified in the present study, particularly for the regions New 1, 2, 3m, and Old 2. Overall, this correlation reaches a level of 68%.
- Two large ground subsidence areas in the western part of the DInSAR map, just south of the offshore New 5 and New 6 regions, are noteworthy. We speculate that these two subsidence regions, although located some distance from the predicted land subsidence regions, may be caused by underground mining activities.
- Note that land subsidence is a slow process, which usually occurs over several years. Hence, the DInSAR map produced from SAR data captured 6 months apart in 2011 shows just a snapshot of the land subsidence-in-progress.
- The developed software allows for producing a landslide susceptibility map, which may support decision-making processes for town and country planning, as well as the development of mitigation strategies for protecting people and technical infrastructure in areas where underground mining operations have increased the risk of landslides.

## Electronic supplementary material

ESM 1 (PY 3 kb)

ESM 2 (TBX 19 kb)
